# Pneumatic compression devices for in-home management of lymphedema: two case reports

**DOI:** 10.1186/1757-1626-2-6625

**Published:** 2009-03-23

**Authors:** Samantha Cannon

**Affiliations:** 1 Gwinnett Sports Rehabilitation, Gwinnett Medical Center, 500 Medical Center Blvd, Suite 130, Lawrenceville, GA 30045

## Abstract

The two patients in this case series had experienced long-term difficulty controlling lymphedema at home. Both patients had used numerous home therapies, including older-generation intermittent pneumatic compression devices, without success. The Flexitouch® system, an advanced pneumatic device, was prescribed to assist them with in-home efforts by providing therapy to their affected limbs in addition to the lower trunk area for the patient with lymphedema of the lower extremity; and the trunk, chest wall, and shoulder areas for the patient with lymphedema of the upper extremity. Both patients achieved successful home maintenance of lymphedema, as judged by limb volume, clinical observations, and subjective patient impressions, after incorporating the Flexitouch® system. Neither patient experienced the deleterious effects (worsening genital edema; fibrotic cuff development) that they had experienced with the older-generation intermittent pneumatic compression devices they had previously used. Incorporating the Flexitouch® system as part of maintenance may improve success for lymphedema patients who have previously struggled with in-home management.

## Introduction

Methods for the home management of lymphedema include self-manual lymphatic drainage (MLD) [1,2], but some patients are not able to perform self-MLD successfully at home due to limited mobility [3], inability to execute the techniques properly, or lack of caregiver assistance. Use of repeated cycles of intensive complete decongestive therapy (CDT) is not a practical long-term solution and is often not covered by insurance [3]. Patients become frustrated with the inadequacy of efforts to control lymphedema [4]. The condition is associated with an adverse psychological impact and impairments in quality of life [3,5].

As a therapist, I eventually became frustrated by barriers to successful home care and began to seek options to improve my patients' likelihood of success. One option to assist in the self-MLD portion of home care for lymphedema is the use of an intermittent pneumatic compression (IPC) device. The National Lymphedema Network Position Statement and the International Society of Lymphology identify IPC as an adjunct to lymphedema therapy, but caution that its use may lead to complications [2,6].

I do not typically recommend compression pumps for lymphedema patients because of negative patient experiences with older-generation pumps. However, an advanced device is now available that is designed to overcome the therapeutic barriers of traditional IPC devices. The Flexitouch® (FT) system (Tactile Systems Technology, Inc, Minneapolis, MN) is a pneumatic device for home maintenance lymphedema therapy [7].

Two cases are presented of patients who had experienced long-term difficulty controlling lymphedema at home. They had previously used traditional IPC without success. The FT system was prescribed to assist in self-care by providing limb treatment in addition to treatment of the lower trunk area for the patient with lower-extremity lymphedema and the chest wall, trunk, and shoulder areas for the patient with upper-extremity lymphedema.

Both patients had required multiple rounds of CDT at different clinics and suffered complications secondary to older-generation pump use prior to presentation at this clinic.

Patients were interviewed about their experiences with older-generation IPC devices and the FT system. In addition, each patient returned to the clinic for two sessions that were approximately one week apart. Measurements of the affected limb were taken with a Juzo® tape measure (Juzo, Cuyahoga Falls, OH) before and immediately after treatment with the FT system at one visit and again after treatment with the IPC devices they had previously used at the next. Total limb volumes were calculated from the measurements.

## Case Presentation

### Case report 1

**Figure 1:**
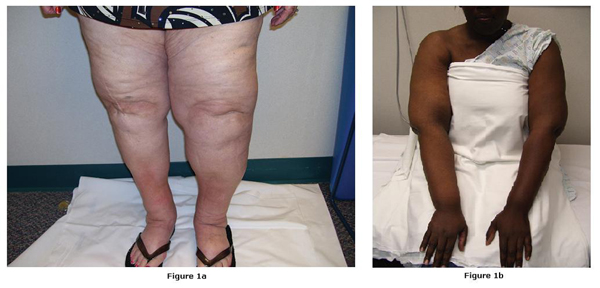
**Patient photographs (A) Illustrating bilateral lower extremity lymphedema greater on the left**. **(B)** Patient 2, showing right upper extremity lymphedema.

The first patient (PT1) was diagnosed in March 2002 with bilateral (left greater than right) lower extremity and genital lymphedema (Figure 1A) secondary to vulvar cancer. A white female non-smoker, when first seen at the clinic she was 75 years old, 222 lbs in weight and 5–1" in height. Her cancer treatment included hysterectomy and excision of 19 inguinal nodes. In 1993, she had a right total knee replacement for arthritis. Her lymphedema treatment began in North Carolina in April 2002. From that time until December 2005, she applied bilateral bandages and used a non-programmable Extremity Pump® System 7500 (Kinetic Concepts, Inc, San Antonio, TX; HCPC code E0651; previously known as the Jobst pump) for lymphedema in her left lower extremity. After three years, she discontinued using the device due to worsening genital edema, lack of efficacy, and difficulty donning the appliances. She reported that the device created increased pressure on the legs and aggravated discomfort at the groin, stating that the device was "too strong" and that it "hurt".

In October 2006, she relocated to Georgia and began treatment at Gwinnett Medical Center for increased pain, decreased range of motion, cellulitis, and fibrosis. She received intensive CDT and HydroTrack^TM^ (Conray, Inc, Phoenix, AZ) therapy, but was unable to perform self-MLD at home due to arthritis and lack of home care assistance. Because this patient required an additional home therapy component but was at risk for exacerbating existing genital lymphedema, in November 2007 she was prescribed the FT system with trunk treatment rather than an IPC that could treat only the limb. Her treatment protocol consisted of one 60-minute treatment session for the trunk and leg per day.

**Figure 2:**
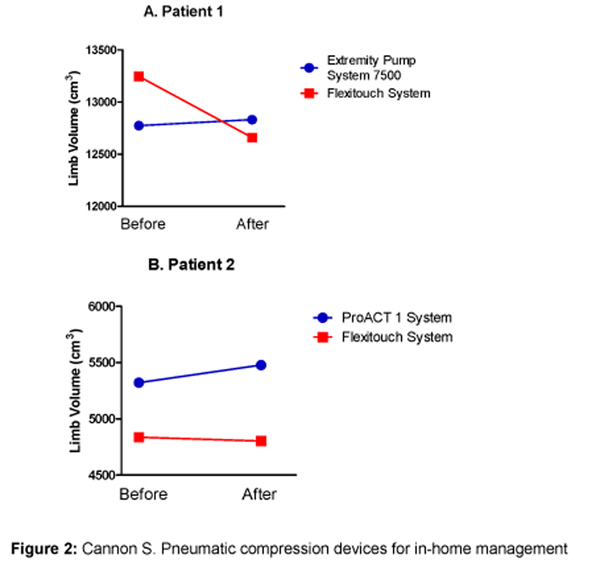
**Measurements were made with a Juzo tape measure**. Lower volume measurements **(A)** showing left lower extremity volume measured before and after a single session of treatment with Flexitouch and with the Extremity Pump System 7500. **(B)** showing right upper extremity volume measured before and after a single session of treatment with Flexitouch and with the ProACT 1 system.

A single in-clinic session with FT resulted in a 4.4% decrease in treated leg volume. By contrast, during a single in-clinic session with the IPC device she had previously used, her treated leg volume increased by 0.45% (Figure 2A).

During her long-term usage of FT, PT1 found the system to be comfortable and easy to apply and remove without assistance, and stated that it was relaxing enough that she could fall asleep during a session. She described urinary urgency occurring immediately after treatment with the FT system, which may be indicative of effective lymphatic drainage. She also stated that she had more stamina, leading to a more active lifestyle, which she attributed to the FT system. She proclaimed that she "could not live without it." At the time of this report, PT1 had used FT for approximately one year; during that year she had no return visits for CDT and experienced none of the complications that had previously developed with use of older-generation IPC devices.

### Case report 2

The second patient (PT2), a black female, was a 43-year-old non-smoker with weight of 300 lbs and height of 6–2" at the time she presented for care at the clinic. She was diagnosed in New York in 2000 with right upper extremity lymphedema (Figure 1B) following treatment for breast cancer in 1998 including mastectomy, axillary dissection of 24 lymph nodes, radiation, and chemotherapy. A side effect of the radiation was fibrosis of the lateral chest wall and breast tissue. The patient's first CDT began in 2002 after the lymphedema progressed with increased discomfort and recurrent infections. Between 2002 and 2006, she had four episodes of cellulitis treated with multiple antibiotics. She received three courses of intensive CDT, including two to three visits weekly for three months. In 2004, she began using the programmable ProACT 1 pump (Fore Tech Medical, Williamstown, NJ; HCPC code E0652) intermittently for temporary relief of pain when her symptoms were most bothersome. This device did not provide treatment of her truncal lymphedema.

In 2006, she stopped using the ProACT 1 pump due to development of a fibrotic cuff (at the limb root nearest the axilla), skin irritation, and lack of sustained reduction of lymphedema. She then moved to Georgia and in March 2007 began a fourth course of CDT, which required treatment five days a week for four months. At the time she began this treatment, arm volume measurements showed that the affected arm had 31% greater volume than the unaffected arm.

The patient's self-MLD was not successful, because she was unable to reach all affected areas and did not have anyone at home to assist with her care. The FT system was recommended because, in addition to treating her affected arm, it treated the chest wall, trunk, and shoulder areas that the patient could not reach. In December 2007, she began using FT for in-home treatment. The treatment protocol with the FT device consisted of one full upper-extremity and chest treatment session per day. A single session with the ProACT 1 pump produced a 2.9% increase in the patient's arm volume (Figure 2B). A session with FT was associated with a 0.68% decrease in arm volume.

During her long-term experience with FT, PT2 reported the device to be comfortable and easy to use without assistance. She experienced softening of the fibrosis in her arm, breast, and chest wall after incorporating the FT system at home. The FT system also effectively treated multiple affected areas, reducing edema in her fingers, palm, forearm, upper arm, shoulder, and back. In addition, with FT usage she did not develop a fibrotic cuff or experience the skin irritation that had previously occurred with usage of the older generation IPC.

Prior to treatment, PT2 received disability benefits due to pain from the lymphedema and swelling. After treatment with FT, she was able to return to work. She has not required additional in-clinic CDT since initiating FT therapy one year ago.

## Discussion

It can be difficult for clinicians to identify effective ways to help lymphedema patients successfully manage home care. Risks associated with ineffective therapy include worsening of the lymphedema, recurrent infections, pain, and reduced range of motion [4,8]. Daily treatment regimens are physically and emotionally demanding, and the patient's quality of life is impaired by both the lymphedema and the rigors of self-treatment [3,9]. Recommended treatment should be both effective and acceptable to the patient.

Both patients in this case series reported discomfort with previously used IPC devices, neither of which provided sustained management of lymphedema. Both had required multiple intensive treatments for lymphedema prior to being seen at Gwinnett Medical Center. Furthermore, these patients' use of older-generation IPC devices had resulted in complications, including worsening genital edema (PT1) and development of a fibrotic cuff (PT2). After incorporating long-term home usage of the advanced FT device, both patients were able to successfully control lymphedema without aggravating complications they had previously experienced. Both patients achieved successful home maintenance of lymphedema, as judged by limb volume, clinical observations, and subjective patient impressions, after incorporating the FT system.

The design features of the FT system may improve patient outcomes and enhance acceptance and tolerance of the device. The device uses a calibrated, work-and-release inflation/deflation pattern applying therapeutic pressures that are significantly different from those produced by older-generation IPCs [10]. The FT system's programmability allows for focus on fibrotic areas and provides treatment to the trunk and chest in addition to the affected limb(s) [7], features that address concerns and limitations expressed about older-generation IPC devices.

## Conclusion

Incorporating the FT system as part of maintenance may improve success for lymphedema patients who have previously struggled with in-home management. Differences in design between the FT system and older-generation IPC devices, as well as the unique means by which the FT system provides truncal therapy, may contribute to beneficial results and generate new interest from therapists and patients in this advanced technology.

### Patient's Perspectives

#### Patient 1

I have lymphedema in both of my legs. Doing treatment at home has been hard. I have arthritis, and I am unable to put on compression garments myself or do self-massage, because I can't reach where I need to. I used an older pump at home to treat my legs. That device was hard to get on and it hurt, even on the lowest pressure setting. It did not help with my fibrosis and in general, was not effective. The Flexitouch is easy for me to get on and off without help. It treats my legs and abdomen. It is soothing and relaxing enough to fall asleep during treatment. The Flexitouch is the most effective pump therapy I've had. I use it every day. If I don't use it, the swelling around my knees makes the arthritis so painful that I can hardly walk. By using the Flexitouch I am able to keep moving. I could not live without it!

#### Patient 2

I got lymphedema after being treated for breast cancer. It affected my arm as well as my chest. I am right-handed and an accountant. Having lymphedema in my right arm caused difficulty with typing and writing. It was painful. I received in-clinic treatment for my lymphedema and was trained to do treatments at home, such as lymphedema massage. But I was not able to reach all affected areas, so I did not see many results from my self-massage efforts. I had tried a different pump in the past on a daily basis but felt only temporary relief of some discomfort while it was on. It did not last, and it had no effect on my edema. It also caused irritation at the zipper, and little sores developed.

My therapist recommended the Flexitouch, and I have been using it for about a year. I am now able to treat lymphedema on my own. It is comfortable, treats all of my chest and arm, and feels very much like my therapist's MLD technique. I am very much satisfied with the results I have had using the Flexitouch system. The results have been longer lasting.

## Abbreviations

MLD: Manual lymphatic drainage; CDT: Complete decongestive therapy; IPC: Intermittent pneumatic compression; FT: Flexitouch®.

## Consent

Written informed consent was obtained from the patients for publication of this case series and accompanying images. A copy of the written consent is available for review by the Editor-in-Chief of this journal.

## Competing interests

The author declares no competing interests.

## Authors' contribution

SC performed evaluations and in-clinic treatments, made limb measurements, prepared and provided custom compression garments and recorded clinical observations for the cases described.

## References

[B1] Cohen SR Payne DK Tunkel RS Lymphedema: strategies for management Cancer 2001 92 4 980 987 10.1002/1097-0142(20010815)92:4+<980::AID-CNCR1410>3.0.CO;2-E 11519024

[B2] National Lymphedema Network Position Statement: Treatment

[B3] Ridner SH McMahon E Dietrich MS Hoy S Home-based lymphedema treatment in patients with cancer-related lymphedema or noncancer-related lymphedema Oncol Nurs Forum 2008 35 671 680 10.1188/08.ONF.671-680 18591171

[B4] Morgan PA Franks PJ Moffatt CJ Health-related quality of life with lymphedema: a review of the literature Int Wound J 2005 2 47 62 10.1111/j.1742-4801.2005.00066.x 16722853 PMC7951421

[B5] Pain SJ Purushotham AD Lymphoedema following surgery for breast cancer Br J Surg 2000 87 1128 1141 10.1046/j.1365-2168.2000.01569.x 10971418

[B6] International Society of Lymphology The diagnosis and treatment of peripheral lymphedema Lymphology 2003 36 84 91 12926833

[B7] Tactile Systems, Inc.

[B8] Szuba A Rockson SG Lymphedema: classification, diagnosis and therapy Vasc Med 1998 3 145 156 9796078 10.1177/1358836X9800300209

[B9] Ridner SH Quality of life and a symptom cluster associated with breast cancer treatment-related lymphedema Support Care Cancer 2005 13 904 911 10.1007/s00520-005-0810-y 15812652

[B10] Mayrovitz HN Interface pressures produced by two different types of lymphedema therapy devices Phys Ther 2007 87 1379 1388 17712034 10.2522/ptj.20060386

